# A Case of Phlegmasia Cerulea Dolens

**DOI:** 10.7759/cureus.111232

**Published:** 2026-06-21

**Authors:** Arvin Bozorg Chenani, Ramadan Ahmed, Sarath Vayolipoyil

**Affiliations:** 1 Internal Medicine, Scarborough Hospital, Scarborough, GBR; 2 General Internal Medicine, Scarborough Hospital, Scarborough, GBR; 3 General Medicine, Scarborough Hospital, Scarborough, GBR

**Keywords:** deep vein thrombosis (dvt), phlegmasia cerulea dolens, pulmonary embolism (pe), venous thromboembolism (vte), venous thrombosis

## Abstract

Phlegmasia cerulea dolens (PCD) is a rare but severe manifestation of extensive deep vein thrombosis (DVT) characterized by near-complete venous outflow obstruction, resulting in massive limb swelling, cyanosis, and risk of venous gangrene. Early recognition and prompt management are essential to prevent limb loss and life-threatening complications. A 55-year-old man presented with sudden-onset swelling, pain, and numbness of the left lower limb that began approximately four hours before presentation. The symptoms initially progressed rapidly and then stabilized without significant further deterioration. The patient reported a sensation of skin tightness but denied limb coldness. He was able to move the affected limb, although movement was limited by pain and swelling. Before symptom onset, he had normal mobility and remained fully independent in daily activities. He denied chest pain, dyspnea, or other cardiopulmonary symptoms. His medical history was significant for hypertension, and his brother had a history of DVT. On examination, the left lower limb was markedly swollen, tender, and demonstrated bluish discoloration with tense skin. Capillary refill time was prolonged, and distal pulses were initially diminished before becoming non-palpable. The limb was not cold, and there was no objective sensory deficit, although the patient reported paraesthesia. Laboratory investigations demonstrated leukocytosis and markedly elevated D-dimer levels. Doppler ultrasonography revealed extensive occlusive thrombus involving the left common femoral and superficial femoral veins extending into the iliac vein. Further imaging confirmed extensive iliofemoral DVT with cranial propagation into the infrarenal inferior vena cava and associated segmental and subsegmental pulmonary embolism. Following vascular surgery consultation, a working diagnosis of PCD was made. The patient was started on therapeutic anticoagulation with apixaban. Due to the family history of venous thromboembolism, thrombophilia screening was arranged with plans for repeat testing after three months. An outpatient echocardiogram was also requested.

## Introduction

Venous thromboembolism, including deep vein thrombosis (DVT) and pulmonary embolism (PE), is common in modern medicine. There is significant variability in the severity of presentation, ranging from relatively small DVTs that require no intervention to severe forms, such as phlegmasia cerulea dolens (PCD). PCD is a massive clot burden within the deep veins, particularly involving the more central veins [1].

PCD represents the most severe form of DVT, resulting from extensive thrombosis of major and collateral venous channels. This leads to massive venous congestion, interstitial edema, and compromised arterial inflow due to increased compartmental pressure. Clinically, it presents with a triad of severe pain, swelling, and cyanosis. If untreated, it can progress to venous gangrene, limb loss, and death. Mortality rates have been reported between 20% and 40%, with amputation rates ranging from 12% to 25% [2,3].

This case highlights the importance of early recognition of PCD, a rare but potentially limb- and life-threatening manifestation of extensive venous thrombosis. Clinicians should maintain a high index of suspicion in patients presenting with the characteristic clinical triad of acute severe limb pain, massive edema, and violaceous or cyanotic discoloration. These findings reflect extensive venous outflow obstruction, which may progress to impaired arterial inflow, venous gangrene, compartment syndrome, and death if left untreated.

A thorough physical examination, including comparison with the contralateral limb, remains fundamental in identifying this condition. Early diagnosis and prompt initiation of anticoagulation, with consideration of endovascular or surgical intervention when indicated, are essential to optimize limb salvage and improve clinical outcomes. Important differential diagnoses include acute arterial occlusion, compartment syndrome, cellulitis, necrotizing soft-tissue infection, lymphedema, and uncomplicated DVT. Risk factors include malignancy, hypercoagulable states, major surgery, trauma, and prolonged immobility. Prompt recognition and management are essential to prevent irreversible complications.

## Case presentation

A 55-year-old man presented with a four-hour history of sudden-onset swelling, pain, and numbness in the left lower limb. Symptoms progressed rapidly initially and then plateaued without further deterioration. He described a sensation of tightness in the affected limb but denied coldness. Although movement was preserved, it was limited by pain and swelling. The patient was previously fully independent with normal mobility. He denied chest pain, dyspnea, or other cardiopulmonary symptoms. His past medical history included hypertension. Family history was notable for DVT in his brother.

**Figure 1:**
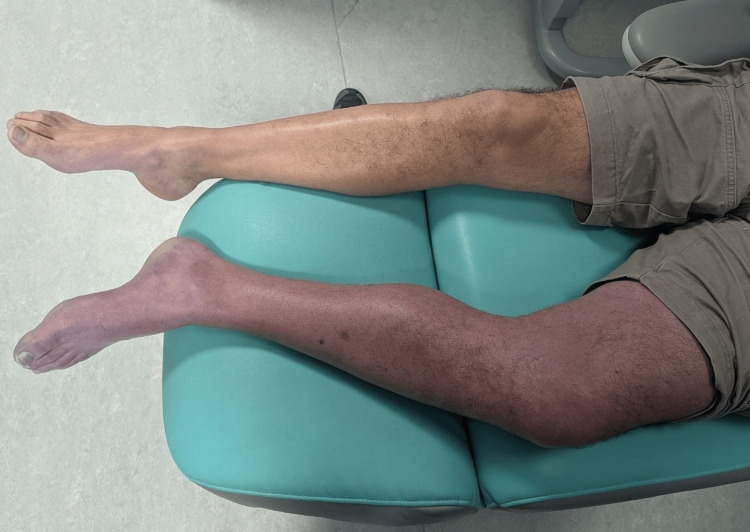
Marked swelling, cyanosis, and tense edema of the left lower limb consistent with phlegmasia cerulea dolens.

**Figure 2:**
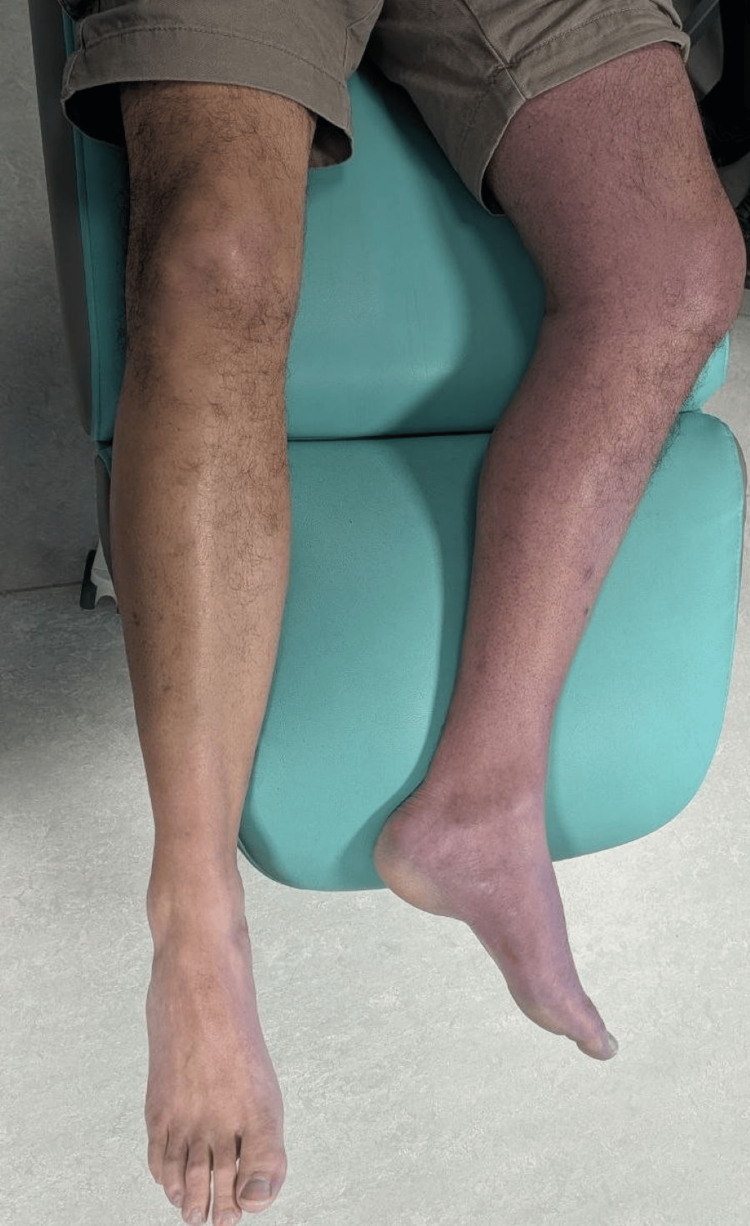
Marked swelling, cyanosis, and tense edema of the left lower limb consistent with phlegmasia cerulea dolens.

On examination, the left lower limb was markedly swollen, tense, and tender, with a bluish discoloration consistent with cyanosis (Figures 1, 2). Capillary refill time was prolonged. Distal pulses were initially weak and later became non-palpable. The limb remained warm, and no objective sensory deficit was detected, although the patient reported paraesthesia.

Laboratory investigations demonstrated leukocytosis with a white blood cell count of 15.03 × 10⁹/L (reference range: 4.0-11.0 × 10⁹/L) and neutrophilia of 11.71 × 10⁹/L (reference range: 2.0-8.0 × 10⁹/L). D-dimer was markedly elevated at 35,200 ng/mL (reference range: 0-550 ng/mL), while C-reactive protein was mildly raised at 12 mg/L (reference range: 0-5 mg/L) (Table 1).

**Table 1:** Laboratory investigations on admission.

Parameter	Result	Reference Range
Hemoglobin (g/L)	167	130–180
White blood cell count (×10⁹/L)	15.03	4.0–11.0
Neutrophils (×10⁹/L)	11.71	2.0–8.0
Platelets (×10⁹/L)	273	150–450
Urea (mmol/L)	7.0	2.5–7.8
Creatinine (µmol/L)	117	59–104
Estimated glomerular filtration rate (mL/minute/1.73 m²)	60	>60
Sodium (mmol/L)	134	133–146
Potassium (mmol/L)	5.6	3.5–5.3
C-reactive protein (mg/L)	12	0–5
D-dimer (ng/mL)	35,200	0–550

**Figure 3:**
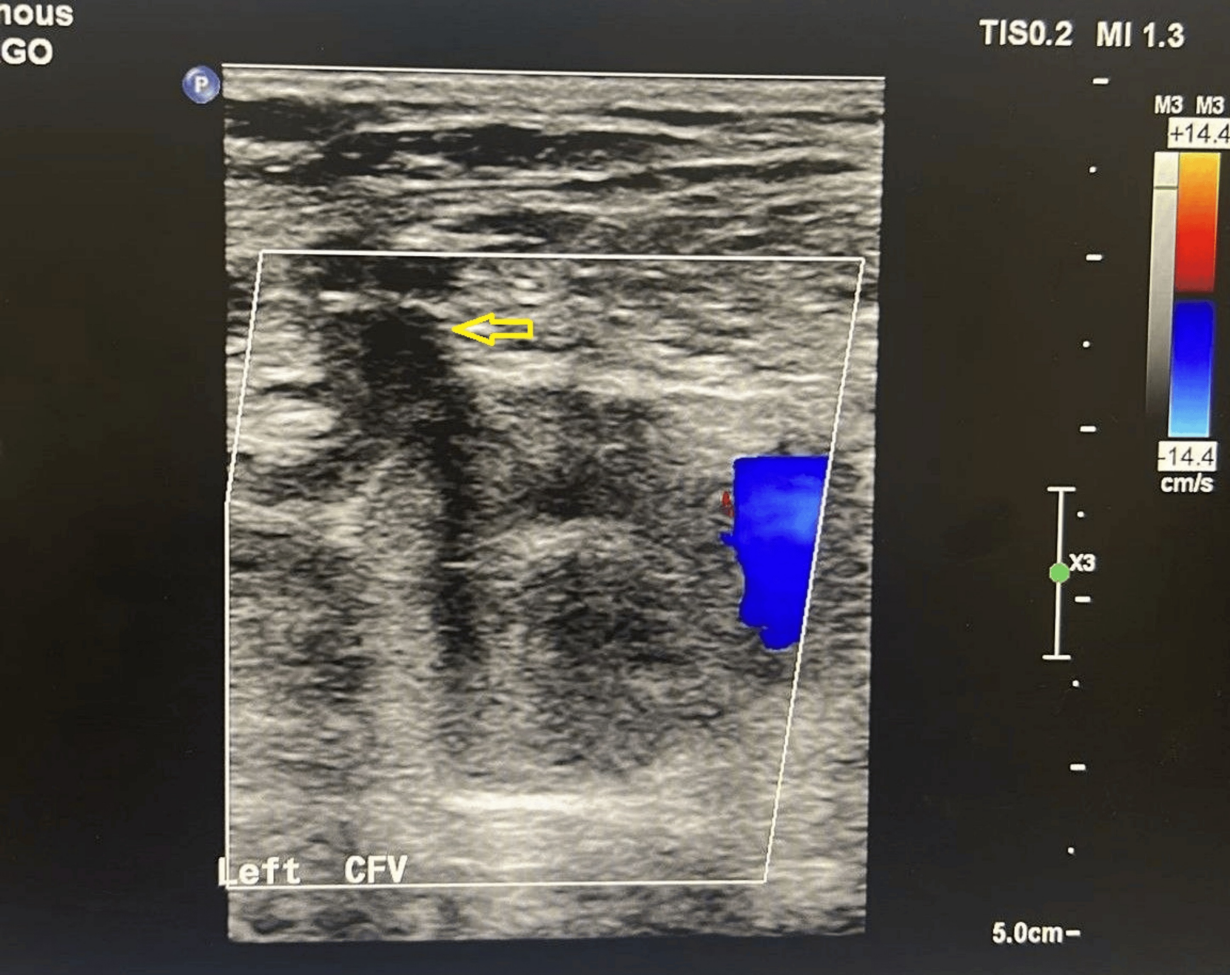
Duplex ultrasonography. Color Doppler image of the left common femoral vein (CFV) showing echogenic thrombus filling the vessel lumen with absent central color signal, confirming occlusive thrombosis.

**Figure 4:**
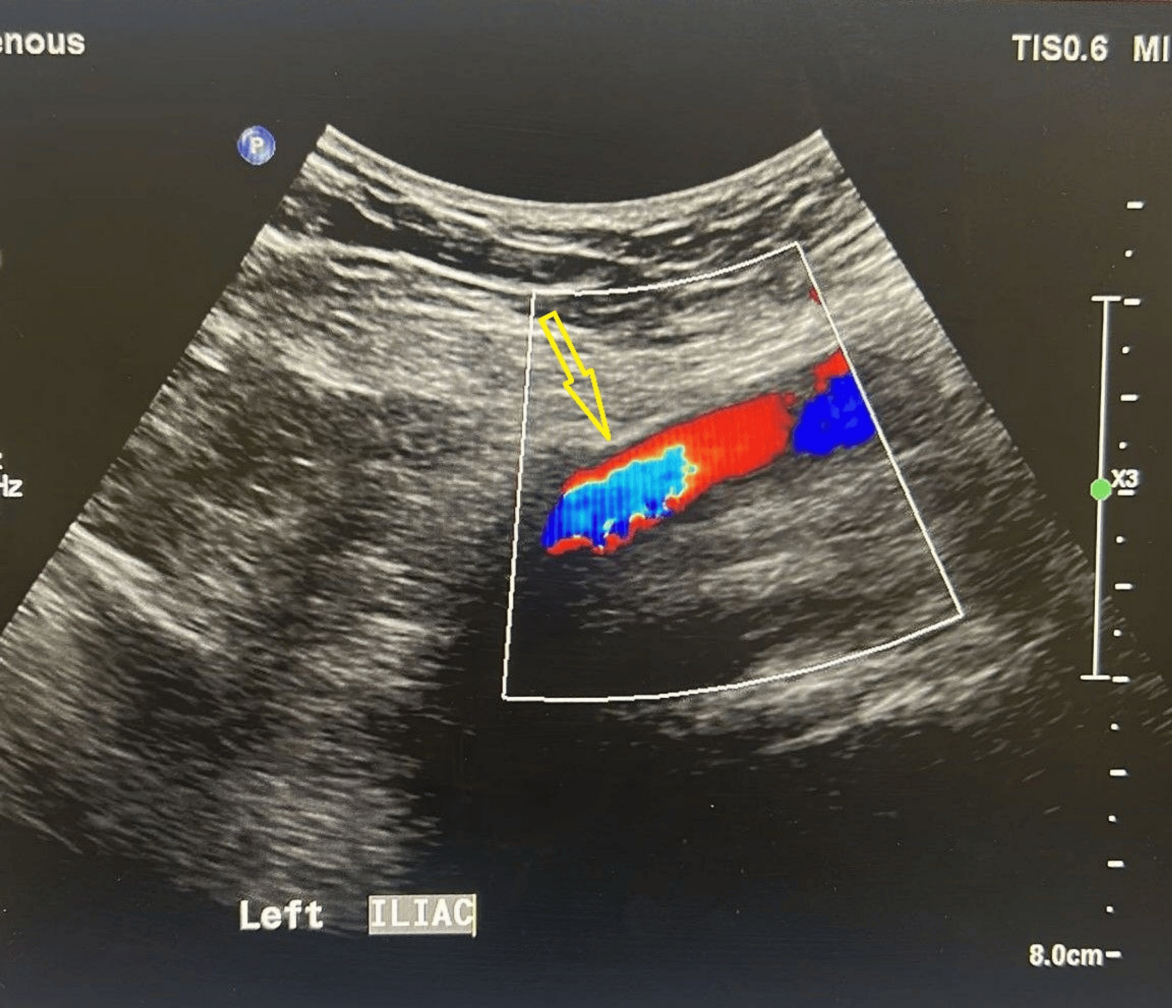
Duplex ultrasonography. Color Doppler image of the left iliac vein showing intraluminal echogenic material with absent central color fill, representing extensive occlusive thrombus extending into the left iliac system.

**Figure 5:**
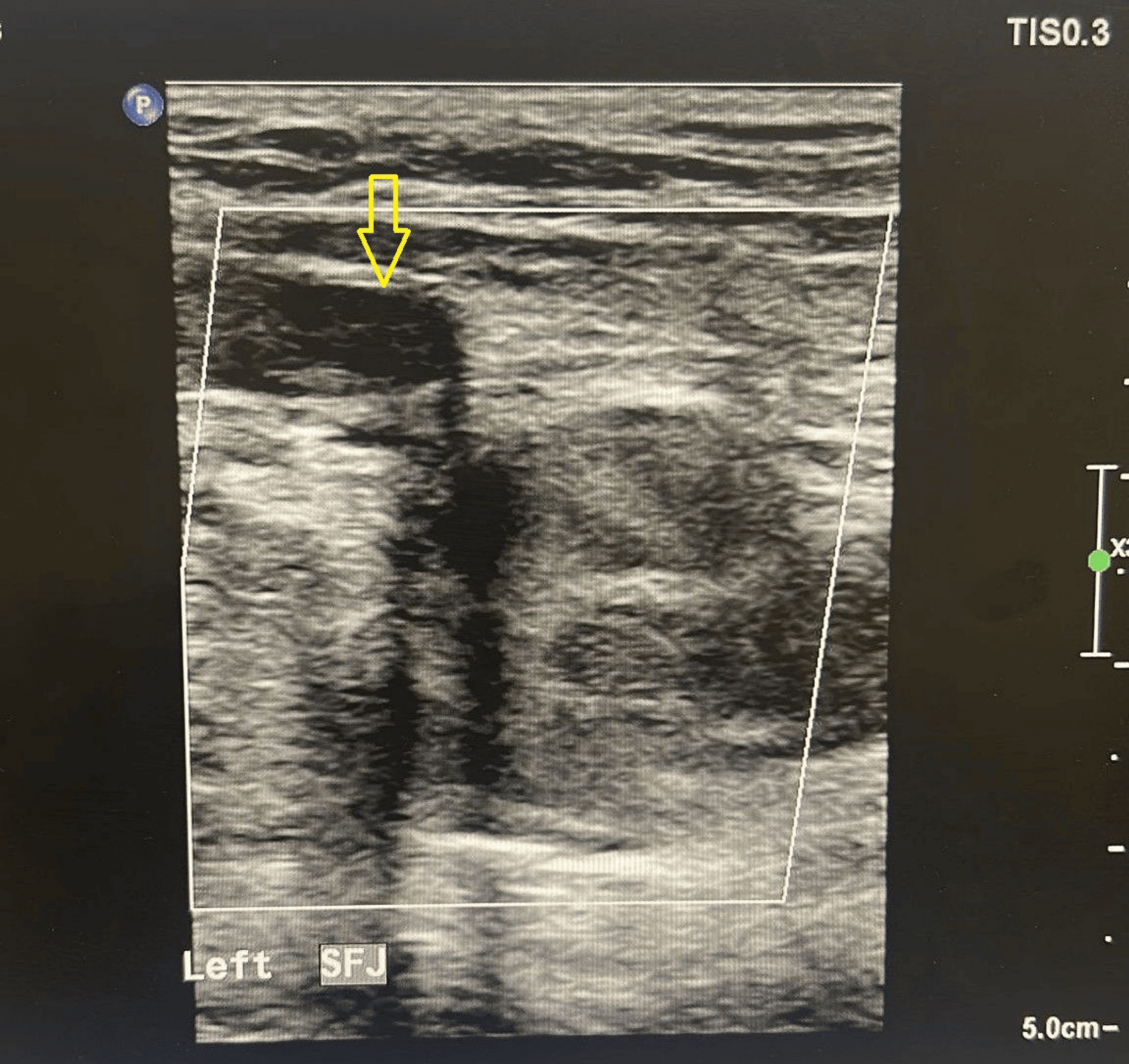
Duplex ultrasonography. B-mode image of the left sapheno-femoral junction (SFJ) showing distended, hypoechoic non-compressible lumen indicating thrombus extension into the SFJ, with no demonstrable color flow.

**Figure 6:**
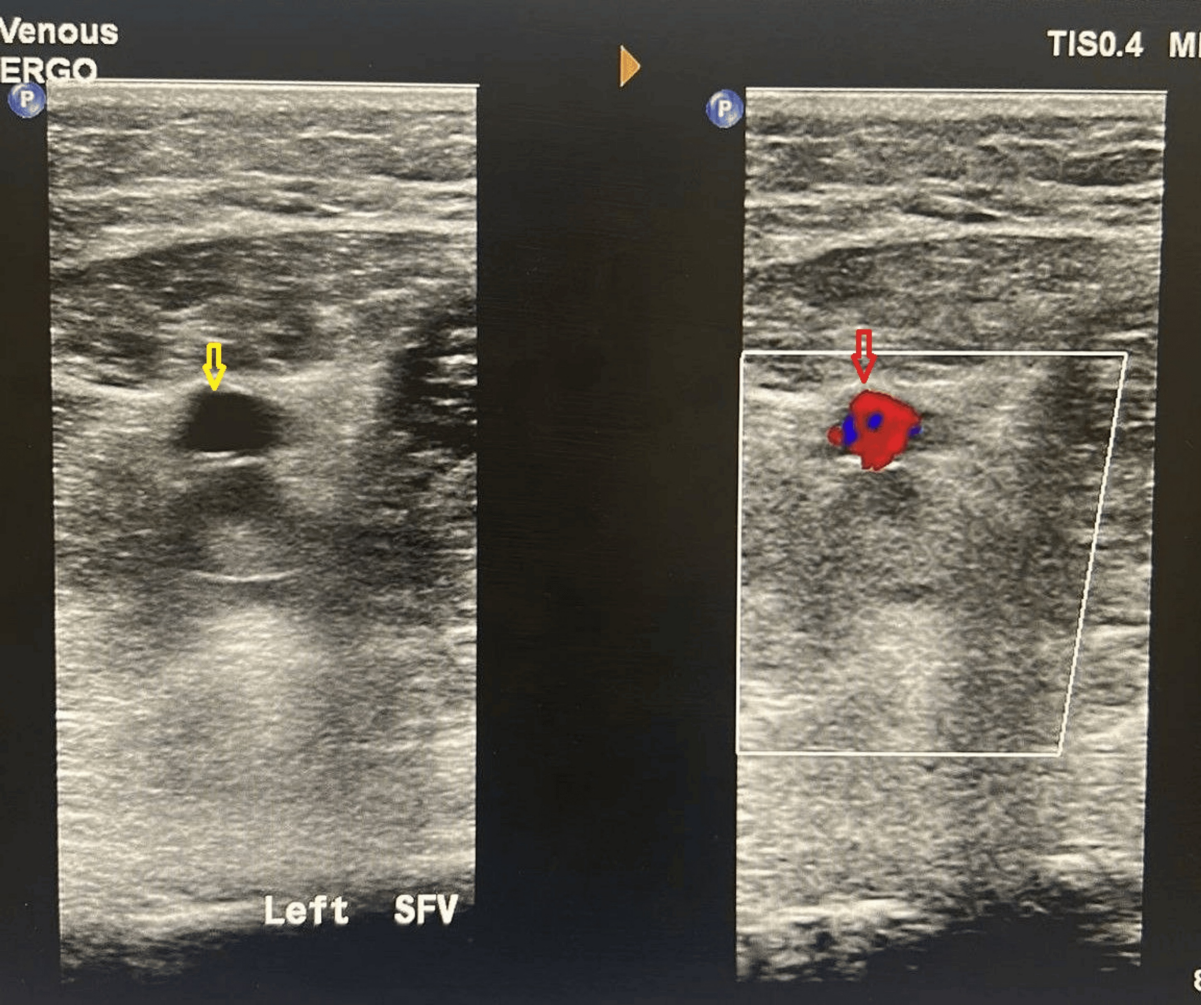
Duplex ultrasonography. B-mode (left panel) and color Doppler (right panel) duplex ultrasound images of the left superficial femoral vein. Yellow arrow: hypoechoic, non-compressible intraluminal thrombus on B-mode, consistent with acute occlusive deep vein thrombosis. Red arrow: absence of color Doppler signal within the lumen confirming complete occlusion.

Doppler ultrasonography demonstrated extensive occlusive thrombus involving the left common femoral and superficial femoral veins, extending into the iliac vein. Cross-sectional imaging confirmed extensive iliofemoral thrombosis with cranial extension into the infrarenal inferior vena cava (Figures 3-6).

Following discussion with the vascular surgery team, a working diagnosis of PCD was made. Further imaging with CT of the thorax, abdomen, and pelvis, CT angiography, and venography revealed bilateral segmental and subsegmental PE without evidence of large central emboli. Imaging also confirmed extensive left iliofemoral DVT extending to the femoral and profunda vein with cranial extension into the infrarenal inferior vena cava. The patient was commenced on therapeutic anticoagulation with apixaban. Thrombophilia screening was arranged because of the family history of venous thromboembolism, with plans for repeat testing after three months. An outpatient echocardiogram was requested, and the patient was monitored for clinical deterioration, including worsening ischemia and development of compartment syndrome.

## Discussion

The management of PCD remains challenging and depends on the severity of venous obstruction, the presence of limb-threatening ischemia, and the patient’s overall clinical status. Given the absence of overt limb ischemia or established gangrene, conservative management with anticoagulation was deemed appropriate in this case [2]. Due to the family history of venous thromboembolism, thrombophilia screening was arranged, with plans to repeat testing after three months to avoid confounding by the acute phase [3].

PCD is a vascular emergency arising from extensive venous thrombosis causing near-total occlusion of venous outflow. This leads to increased venous pressure, massive edema, and reduced arterial inflow, which may ultimately result in tissue ischemia and gangrene [4]. The condition is often associated with underlying malignancy or hypercoagulable states, although it may also occur in patients with more common risk factors such as immobility or inherited thrombophilia [4,5]. In this case, the presence of a positive family history raised suspicion for a possible underlying thrombophilic disorder.

Clinically, PCD is characterized by the triad of pain, swelling, and cyanosis. The presence of preserved warmth in early stages helps differentiate it from arterial occlusion; however, progression may lead to phlegmasia alba dolens or frank venous gangrene [4]. Diagnosis is primarily based on imaging, with duplex ultrasonography serving as the initial modality. CT or MR venography can further delineate the extent of thrombus and involvement of proximal veins and the inferior vena cava.

Management strategies depend on severity and may include systemic anticoagulation, catheter-directed thrombolysis, pharmacomechanical thrombectomy, or surgical venous thrombectomy. Catheter-directed thrombolysis and pharmacomechanical thrombectomy may be considered in selected patients with extensive iliofemoral thrombosis, particularly when there is threatened limb viability, severe symptoms, or failure of anticoagulation alone. These approaches aim to reduce thrombus burden, restore venous patency, and potentially decrease the risk of post-thrombotic syndrome. However, treatment decisions should be individualized, taking into account the severity of ischemia, bleeding risk, comorbidities, and local expertise. In our patient, the absence of progressive limb ischemia, venous gangrene, or compartment syndrome supported a conservative approach with anticoagulation alone. In severe cases with compartment syndrome, fasciotomy may be required [5]. Early anticoagulation remains the cornerstone of treatment and is essential in preventing thrombus propagation and embolic complications [2]. In this patient, anticoagulation alone was appropriate given the absence of limb-threatening ischemia.

## Conclusions

PCD is a rare but life-threatening complication of extensive DVT requiring urgent recognition and management. This case highlights the importance of early diagnosis, prompt anticoagulation, and appropriate investigation for underlying risk factors. Timely intervention can prevent progression to venous gangrene, limb loss, and death.
